# Environmental Variability and Cyanobacterial Blooms in a Subtropical Coastal Lagoon: Searching for a Sign of Climate Change Effects

**DOI:** 10.3389/fmicb.2018.01727

**Published:** 2018-07-31

**Authors:** Márcio S. de Souza, José H. Muelbert, Luiza D. F. Costa, Eliana V. Klering, João S. Yunes

**Affiliations:** Instituto de Oceanografia/Universidade Federal do Rio Grande, Rio Grande, Brazil

**Keywords:** harmful algal blooms, *Microcystis*, wind-driven hydrodynamics, annual rainfall, interannual variability, Patos Lagoon

## Abstract

Cyanobacterial blooms in marine and freshwater environments may be favored by shifts in physical water column parameters due to warming under climate change. The Patos Lagoon (PL), a subtropical coastal environment in southern Brazil, is known for recurrent blooms of *Microcystis aeruginosa* complex (MAC). Here, we analyze the variability of these blooms and their relation to changes in wind direction and speed, rainfall and freshwater run-off from 2000 to 2017. Also, we discuss both longer time-series of air temperature and rainfall and a review of local studies with microcystins produced by these noxious species. Since the 1980s, MAC blooms were associated to negative anomalies in annual precipitation that occur during La Niña periods and, in the last years (2001–2014), accompanied by a trend in low river discharge. MAC blooms were conspicuous from December to March, i.e., austral summer, with massive patches seen in satellite images as for 2017. We suggest that low rainfall and run-off years under NE wind-driven hydrodynamics might accumulate MAC biomass in the west margin of the PL system. In contrast, a positive, long-term trend in precipitation (from 1950 to 2016; slope = 3.9868 mm/yr, *p* < 0.05) should imply in high river discharge and, consequently, advection of this biomass to the adjacent coastal region. Due to the proximity to urban areas, the blooms can represent recreational and economic hazards to the region.

## Introduction

Increasing trends in frequency and intensity of cyanobacterial blooms in several aquatic systems have been observed worldwide (Paerl and Huisman, 2009; Paerl and Otten, 2013; Cloern et al., 2016; Paerl, 2017). Many cyanobacteria of the genus *Microcystis* Lemmermann 1907, can affect the environment and trigger shifts in trophic structure and dynamics, influencing their competitors, consumers and decomposers (Paerl and Pinckney, 1996; Paerl, 2017). *Microcystis aeruginosa* (Kützing) Kützing 1846 (hereafter referred as to MAC for *Microcystis aeruginosa* complex) can bring about economic issues to the fisheries and production of cultivated animals because they are microcystin producers, a hepatotoxin that can potentially affect invertebrates and some vertebrates (Yunes et al., 1996, 1998a). Interestingly, some new brands of microcystin variants found in benthic cyanobacterial mats from Svalbard archipelago appear to have a neurotoxic potential (Kleinteich et al., 2018). More seriously, harmful cyanobacteria blooms (cyanoHABs) can pose health problems to populations living in cities located near aquatic systems recurrently affected by these biological features (Yunes et al., 1998b; Paerl, 2017).

A recent review has already been shown that these microcystins can be raised in importance through many coastal ecosystems; yet estuarine and marine waters are under-investigated (Preece et al., 2017). For instance, MAC biomass should be advected from freshwater to marine systems as into the San Francisco Bay estuary (Lehman et al., 2005). As well *Microcystis* and other cyanoHABs have been reported since the 1980s in the Baltic Sea (Suikkanen et al., 2013; Preece et al., 2017) under a climate change scenario and shifts of hydrological cycle (IPCC, 2014). CyanoHABs have been strongly related with short-term environmental variations both in space and time scales (e.g., seasonal cycles) (Paerl, 2017 and references therein). As primary producers' uptake tends to lower the nutrient levels within the water bodies and strong water column stratification takes place, cyanoHABs can be favored as seen during summer periods worldwide. Furthermore, nutrient pulses into lagoonal systems and coastal regions, mainly of nitrogenous forms, originated by anthropogenic activities might be relatively higher than the natural inputs with uncertain environmental and economic consequences (Battye et al., 2017). This kind of anthropogenic influence has been associated with the increasing trend of cyanoHABs (Paerl, 2017 and references therein).

The predominance of cyanobacteria in summer periods has been observed in the Patos Lagoon (PL) system, southern Brazil. PL is characterized as a mainly light-limited environment and, sporadically in some winters, co-limited by light and nutrients (Odebrecht et al., 2010a). For instance, the effect of nutrient inputs has been partly associated with anthropogenic eutrophication processes within the estuarine region (Niencheski and Baumgarten, 2007; Haraguchi et al., 2015). However, no studies have investigated the annual frequency and intensity of cyanoHABs in the PL system. And long-term changes in these features can be associated to anthropogenic eutrophication (from agricultural, domestic/urban and industrial sources) or related to natural meteo-hydrological variability in the region (wind, rainfall, river discharge).

These variations can also be linked to global warming trend effects on primary producers. For instance, there is an increasing trend in the relative contribution of dinoflagellates and cyanobacteria to the phytoplankton community in parallel with high diatom growth in the estuarine region of PL system (Haraguchi et al., 2015). Long-term studies (1993–2012) revealed that high rainfall and river discharge periods tend to present very low phytoplankton biomass (Haraguchi et al., 2015), whereas cyanoHABs can be advected from the northernmost parts of PL system to the coastal region (Yunes, 2009). Reports indicate that these limnic cyanoHABs can notably be seen along the coastline close to the outlet channel of PL system, mainly during warmer months (Yunes et al., 1998a). In addition, other studies have observed massive cyanoHABs under La Niña typical conditions (low rainfall and river discharge) (Yunes et al., 1998a; Odebrecht et al., 2005). Conversely, during El Niño when annual precipitation and river discharge is high in the region, low phytoplankton biomass is observed within the PL system (Odebrecht et al., 2010a).

Considering all the information above, this work addresses an environmental characterization of cyanoHABs (related to MAC) under an interannual variability context, in the PL system and presents some other cyanobacteria species found in non-bloom conditions. Additionally, the role played by the meteorological variables wind speed and direction and rainfall, and freshwater run-off on the occurrence of these blooms of MAC is investigated. Lastly, this study discusses the influence of global warming effects on the meteorological interannual variability and over the intensity and temporal variation of MAC blooms in the PL system.

## Materials and methods

### Study area

**Figure 1:**
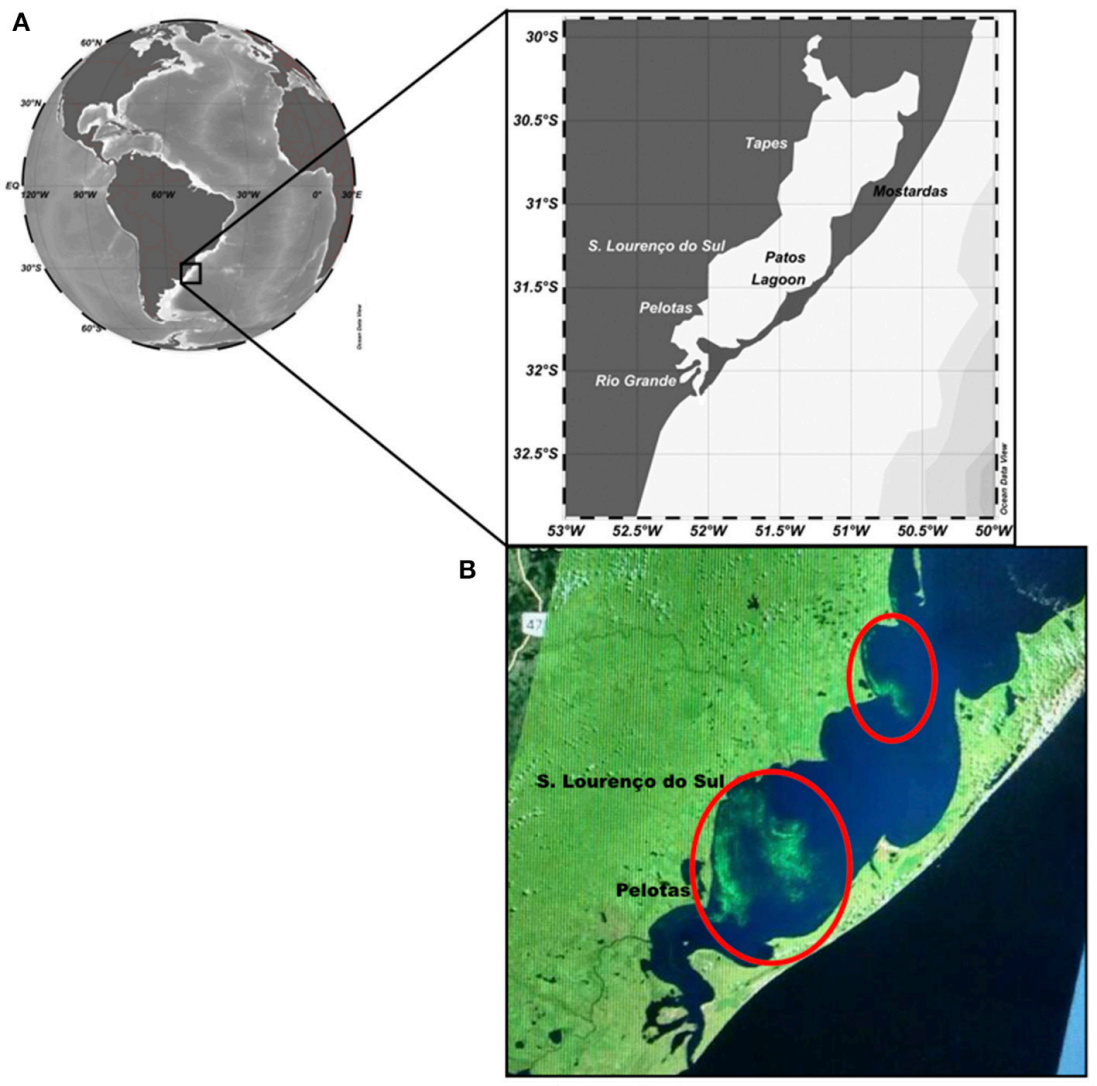
Map of the study area showing **(A)** cities at the west margin of the Patos Lagoon system, where patches (two red ellipses) of *Microcystis* spp. have been notified; **(B)** patches of *Microcystis* spp. (inside of the red circle) seen on 02 February 2017, retrieved from Landsat-8 Enhanced Thematic Map plus (ETM+; https://eros.usgs.gov/satellite-imagery).

The study region is the Patos Lagoon system (30°12′-32°12′S, 050°40′-052°15′W), a subtropical coastal lagoon located in the southernmost part of Brazil (Figure 1). It is the second largest waterbody in Brazil and the largest lagoonal complex in South America. The lagoon waters span 320 km to the north and between 3 and 64 km wide. A drainage basin of approximately 200,000 km^2^ formed by several rivers, provides 75–80% of freshwater to the Patos Lagoon estuary. Lagoon waters are used for drinking supply, fisheries, leisure, navigation, and agriculture and receive domestic and industrial sewage. Dissolved nutrients (from river inputs) transit ~225 km from the north before reaching the estuarine area at the Feitoria Channel (31°41′S and 051°55′W). The sea provides another 10% of water and nutrient input. The São Gonçalo River completes the volume of estuarine waters and also supplies water to houses, industries, and farms in the region. Cyanobacterial blooms of *Microcystis aeruginosa* complex (MAC) are frequent in the estuary waters mainly during summer months. Although the first scientific record dates back to 1987 (Odebrecht et al., 1987), anecdotal evidence of these cyanobacterial blooms across the PL estuary from old local villagers dates back to the beginning of the 20^th^ century (Yunes et al., 1998a). More details on environmental characteristics and hydrology in this region are presented in Yunes et al. (1998a); Odebrecht et al. (2005); Haraguchi et al. (2015) and Abreu et al. (2016).

### Cyanobacteria data

This study uses cyanobacterial bloom (related to MAC) and non-bloom data in an interannual variability context, from few records beginning in the 1980s to summer periods from 2011 to 2017. Available estimates of cyanobacteria composition and/or abundance were obtained from the State Foundation for Environmental Protection Agency (FEPAM; http://www.fepam.rs.gov.br). FEPAM conducts a monitoring program at recreational beaches in the Patos Lagoon during warm periods (November/December to February/March) and after notification of any massive, visible cyanobacteria patches. FEPAM has been estimating cyanobacteria abundance for Tapes (30.66°S, 051.39°W; from 2011 to 2017) and for São Lourenço do Sul (31.38°S, 051.96°W; only from Nov2011 to Feb2012). Sedgewick-Rafter counting chamber was used to determine the abundance of cyanobacteria (cell mL^−1^). As the technical staff from the FEPAM conducted all the morphological identification and counting of these cyanobacteria (e.g., Komárek et al., 2014), we have strictly followed the list of species found for those cities above, even with some species assembled in the genus *Anabaena*. The presence of the benthic cyanobacteria *Anabaena* could be ascribed to the sampling points placed near the margins (<1 m), where resuspension could have mixed planktonic and benthic species. Besides these estimates, data from published studies on cyanobacterial blooms in the region (Yunes et al., 1998a; Odebrecht et al., 2005; Rosa and Garcia, 2013) were revisited in order to draw the relationship between these blooms and meteorological/hydrological parameters in a long-term approach. Moreover, a review of all experimental and field data available of levels/effects of toxins produced by these cyanobacteria, especially of the genus *Microcystis*, are discussed to illustrate their potential threats to ecosystem functions and services to the cities bordering the Patos Lagoon.

### Meteorological and hydrological parameters

A database encompassing multiple sources of meteorological information since 1950, including precipitation and air temperature data, was used in this work for drawing trend lines. Data from a Brazilian meteorological station in the Rio Grande (INMET, Rio Grande) were obtained for the period from 2001 to March 2017 (end of austral summer). Daily river discharge data were obtained from the National Water Agency (http://www2.ana.gov.br) from 2000 to 2014, specifically for the three largest rivers (Jacuí, Taquari, and Camaquã), which together are the major tributaries to the Patos Lagoon (Vaz et al., 2006). We adopted the approach used by Haraguchi et al. (2015), where each monthly mean river discharge was calculated, and the relative contribution of a river to the combined discharge was used to estimate for those missing monthly discharges. Recent data of wind speed and direction (from 2001 to 2013) were also obtained from a meteorological station in the Rio Grande city (near the navigational channel), and used in the free software WRPLOT (https://www.weblakes.com/products/wrplot/) for plotting 4-day, weekly, monthly and summer wind rose diagrams. In order to relate this wind pattern with the summer cyanobacterial patches, wind roses for the austral summer period from December to February were presented. 3.4-ENSO (El Niño Southern Oscillation) index (http://www.esrl.noaa.gov/psd/gcos_wgsp/Timeseries/Nino34/) was used to check at some extent the relationship between hydrological anomalies potentially linked to phytoplankton/cyanobacteria dynamics in the PL system, taking into account interannual variations.

### Statistical analyses

Monthly and annual means were calculated to describe the seasonal and long-term trends of the meteorological and hydrographic variables in the PL system. 3.4-ENSO index anomalies were calculated and divided into La Niña (<0) and El Niño (>0) years for better visualization. In a meteorological context, anomalies assess the deviation in observed values of certain variables in relation to its historical or climatological means. Therefore, we estimated annual anomalies of air temperature and rainfall by the using the following equation: α_*i*_ = *x*_*i*_ - ${\bar{x}}_{i}$ where α corresponds to the anomaly of a certain variable (air temperature or rainfall), *i* corresponds to a particular year, *x* is the meteorological variable of a *i* year $\bar{x}$ and is the mean value of that variable taking into account the whole period of time. The seasonal and interannual variability in air temperature, rainfall, and freshwater discharge were analyzed by plotting respective trend lines. Published data (Yunes et al., 1998a; Odebrecht et al., 2005; Rosa and Garcia, 2013) and recent estimates of cyanobacterial abundance/biomass were overlaid as labels on the rainfall anomaly graph. Recent cyanobacteria abundance data were shown as minimum and maximum values of cell mL^−1^.

## Results

### Meteorological and hydrological parameters

**Figure 2:**
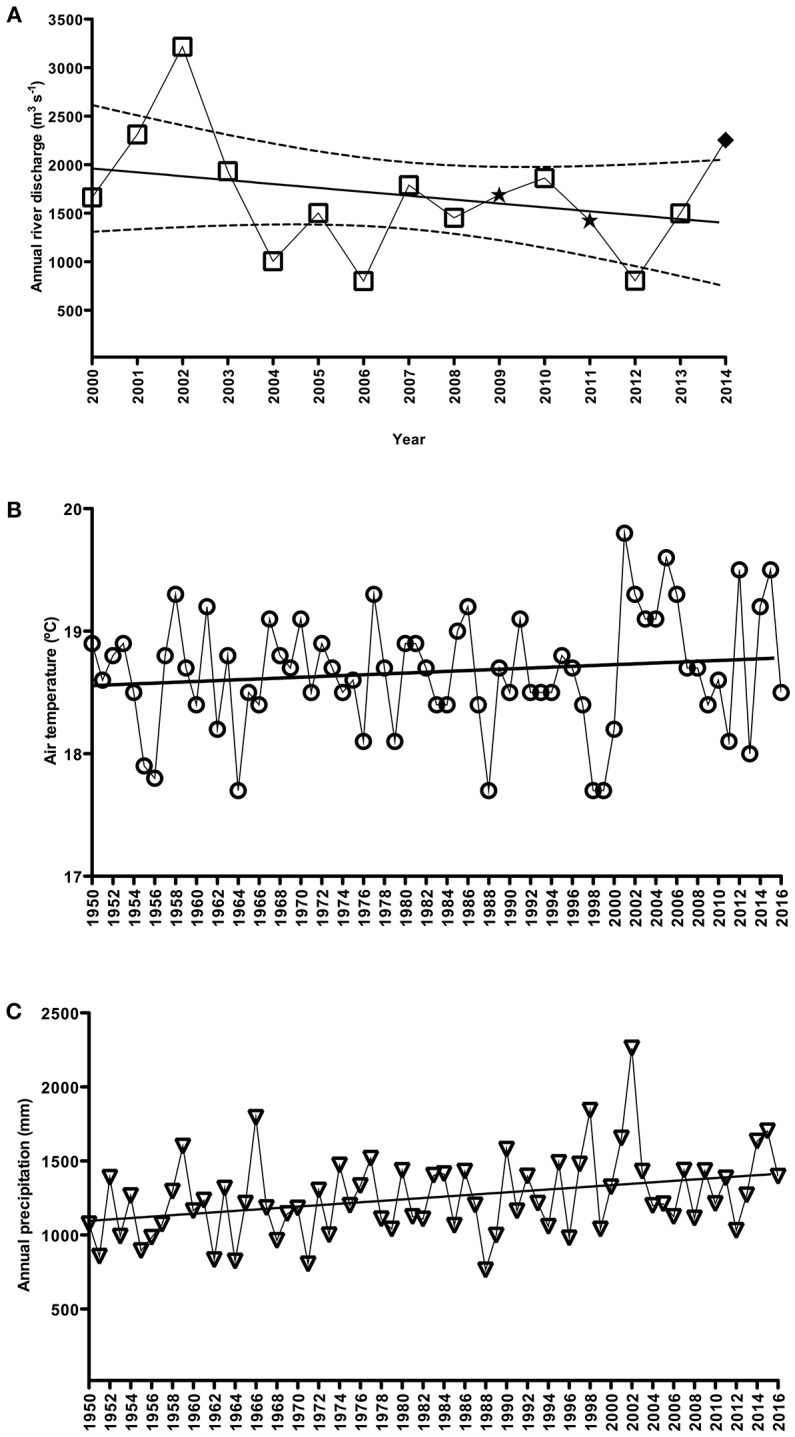
Annual trend (2000–2014) in river discharge (m^3^ s^−1^) **(A)** of three principal rivers (Jacuí, Taquari and Camaquã) for the Patos Lagoon system, and annual trends (1950–2016) for air temperature (°C) **(B)** and accumulated precipitation (mm) **(C)** based on the Rio Grande meteorological station (INMET, Brazil). Note there are missing data for some particular time periods in Figure 2A as follows: in (⋆) August–October 2009 for Jacuí river and October 2009 for Taquari river, and March 2011 for the three rivers; and in (♦) November–December 2014 for Camaquã river. Also, note dashed lines referring to 95% confidence intervals in **(A)**.

**Figure 3:**
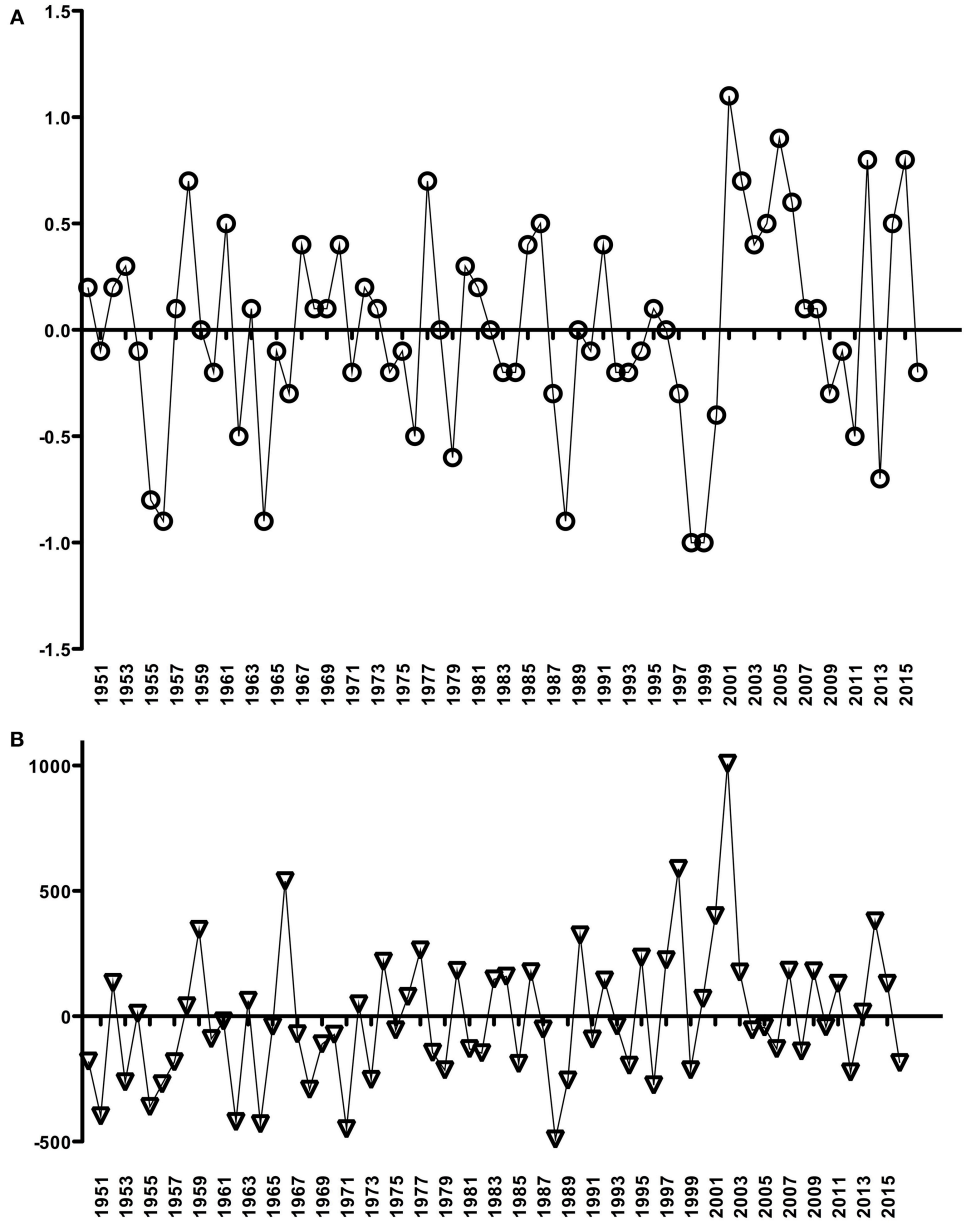
Temperature (°C) **(A)** and accumulated precipitation (mm) **(B)** anomalies, based on annual mean data from the Rio Grande meteorological station (INMET, Brazil), for the Patos Lagoon system. Time intervals for these respective graphs are the same as shown in Figure 2.

**Figure 4:**
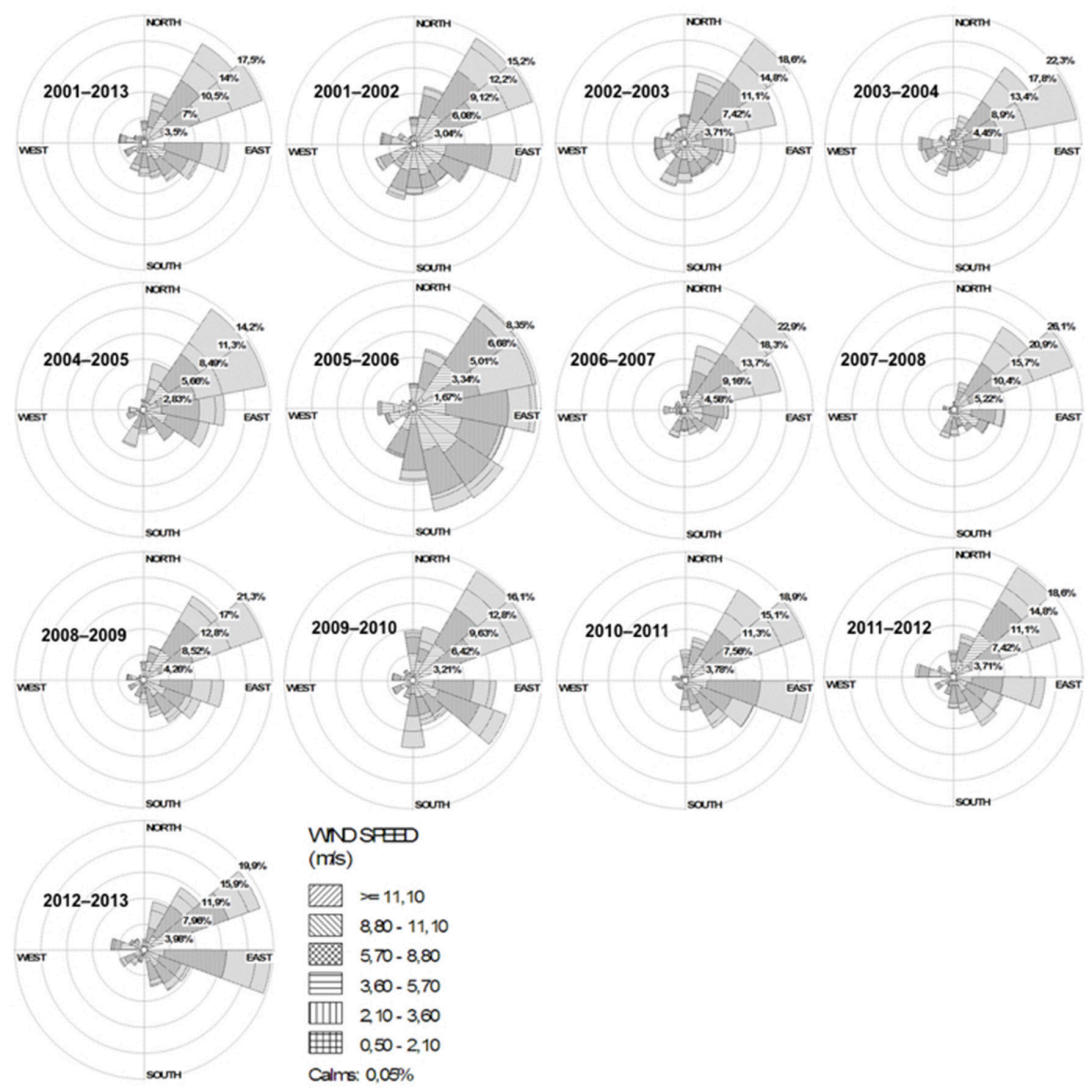
Wind roses chart with wind speed (m s^−1^) and direction, corresponding to the whole time series of 2001–2013 and, in sequence, from December to February, beginning in the austral summer of December-2001 to February-2002 and ending in the austral summer of December-2012 to February-2013. Note that the wind rose from 2005 to 2006 is very dissimilar to others, but having the *N* = 860 of valid data points while the other wind roses have valid data points ranging from 2,130 to 2,190.

A non-significant, decreasing trend in river discharge from 2001 to 2014 was calculated (slope = −0.0296 m^3^s^−1^/yr; *p* = 0.29) (Figure 2A), and low values were generally observed during the summer periods (January to March; data not shown). In contrast, the period from 1950 to 2016 revealed no trend in air temperature (slope = 0.0035°C/yr; *p* = 0.26) (Figure 2B) but a significant positive rainfall trend (slope = 3.9868 mm/yr; *p* < 0.05) (Figure 2C). From 2001 to 2016, some years were highlighted (2001–2008, 2012 and 2014–2015) by positive anomalies and other few years (2009–2011, 2013 and 2016) with negative air temperature anomalies (Figure 3A). Positive precipitation anomalies were observed for 2001–2003, 2007, 2009, 2011 and 2013–2015 (Figure 3B). The years with more pronounced positive anomalies in air temperature and annual precipitation were particularly 2001–2002 and 2014–2015. At the time interval of 2001–2013, the wind rose charts showed a typical pattern of dominant north-easterly winds (~35%) for summer (from December to February next year) reflecting the pattern in the whole period (Fig. 4). This predominance of NE wind ranged from 16.7% in 2005–2006 up to ~47% in 2007–2008 (Figure 4), however, it was noteworthy that the number of valid data points in 2005–2006 was lower (*N* = 860) than in other summer periods (*N* = 2130–2190).

### Relation between cyanobacteria data and environmental parameters

Estimates of *Microcystis aeruginosa* complex (MAC) and/or other cyanobacteria in non-bloom conditions for the summer periods (December–February) of 2011–2017 are presented (Table 1). Blooms were noticed in both cities of Tapes and São Lourenço do Sul at the west margin of PL during the summer period of 2011–2012 with similar concentration (maximum up to 4 × 10^4^ cell mL^−1^). The highest abundance was determined for the summer of 2016–2017 in Tapes only (attaining 2.3 × 10^6^ cell mL^−1^), when there was a bloom of MAC. During this latter period, intense chlorophyll-*a* stripes were visible in an image derived from the Landsat-8 ETM+ but mainly near São Lourenço do Sul and Pelotas (see Figure 1). In the other summer periods, filamentous cyanobacteria were prominent such as *Dolichospermum, Gleiterinema*, and *Limnothrix* along with chroococcaleans (*Merismopedia* and *Microcystis*) denoting a mixed assemblage of cyanobacteria species (Table 1). In the summer of 2011–2012, benthic filaments of *Anabaena* spp. were also noticed.

**Table 1:** Minimum and maximum values of cyanobacteria cells mL^−1^ during warmer months (December–February) near the cities of Tapes and São Lourenço do Sul, with major taxa identified.

**Summer period**	**Tapes**	**São Lourenço do Sul**	
**December–February**	**cell mL**^−1^ **(min-max)**	**cell mL**^−1^ **(min-max)**	**Main taxa**
2011–2012	600–40,000	64–25,000	*Anabaena* spp., *Microcystis aeruginosa* complex (MAC) and other chroococcaleans
2012–2013	0–400	^*^	*Dolichospermum, Geitlerinema, Limnothrix, Merismopedia* and other chroococcaleans and oscilatoriales
2013–2014	18–3,900	^*^	*Dolichospermum, Limnothrix, Merismopedia, Microcystis*
2014–2015	35–150,000	^*^	*Dolichospermum, Limnothrix, Merismopedia, Microcystis*, other chroococcaleans and oscilatoriales
2015–2016	negligible	^*^	
2016–2017	9,300–2,300,000	^**^	MAC

* No data collected for these periods, despite the intense bloom recorded in February 2017 (

** *, see Figure 1)*.

**Figure 5:**
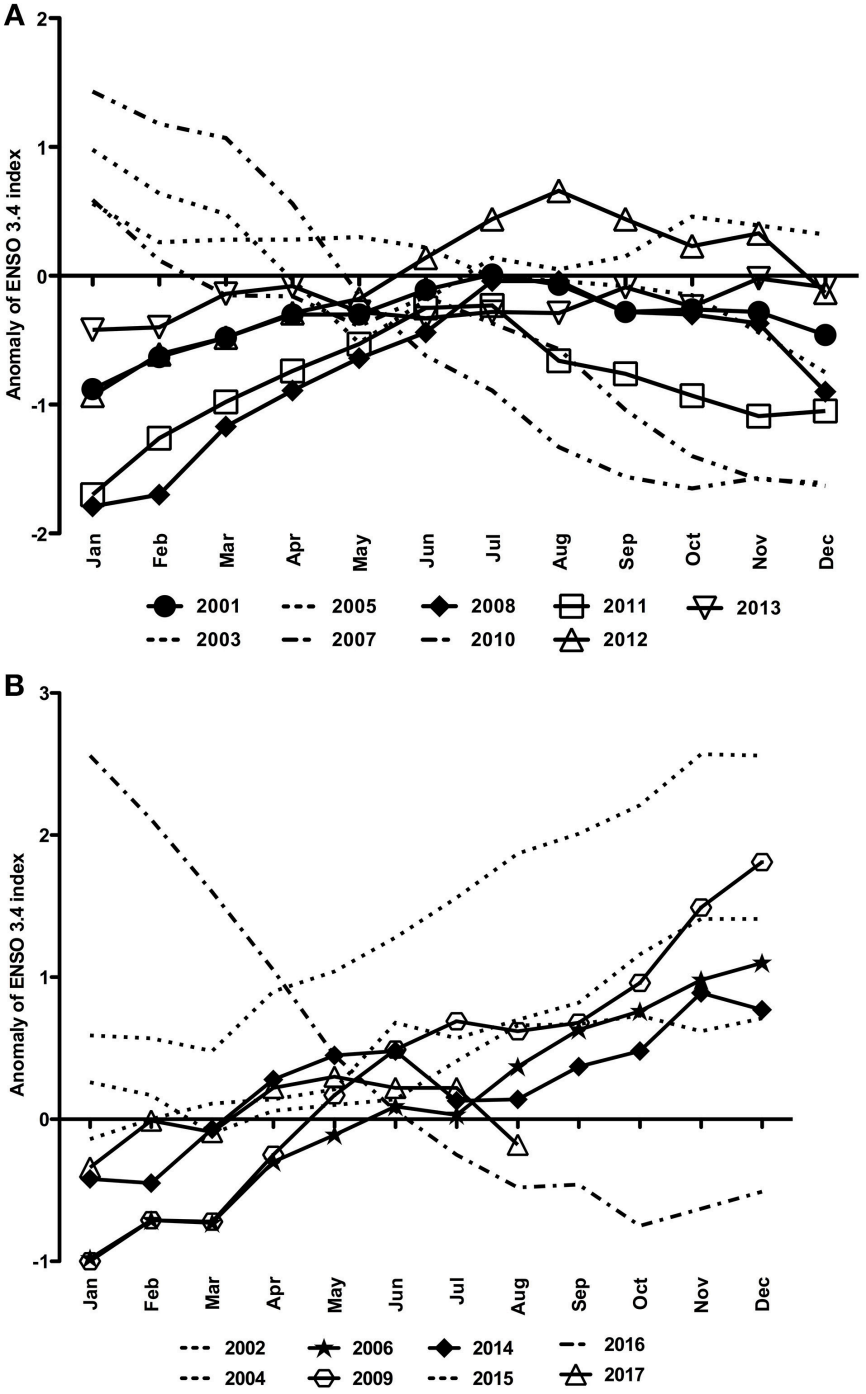
Anomalies of the ENSO3.4 index for the time series of 2001–2017 split into **(A)** La Niña years and **(B)** El Niño years.

La Niña and neutral years were evinced at the end of 2007 and 2010 and in January–February of 2001 and 2012 when there were negative anomalies (Figure 5A). Also, similar negative anomalies were seen in January–March of 2006, 2009, 2014 and 2017 (Figure 5B). On the other hand, the highest positive anomalies characterized the El Niño of 2015–2016 (Figure 5B).

**Figure 6:**
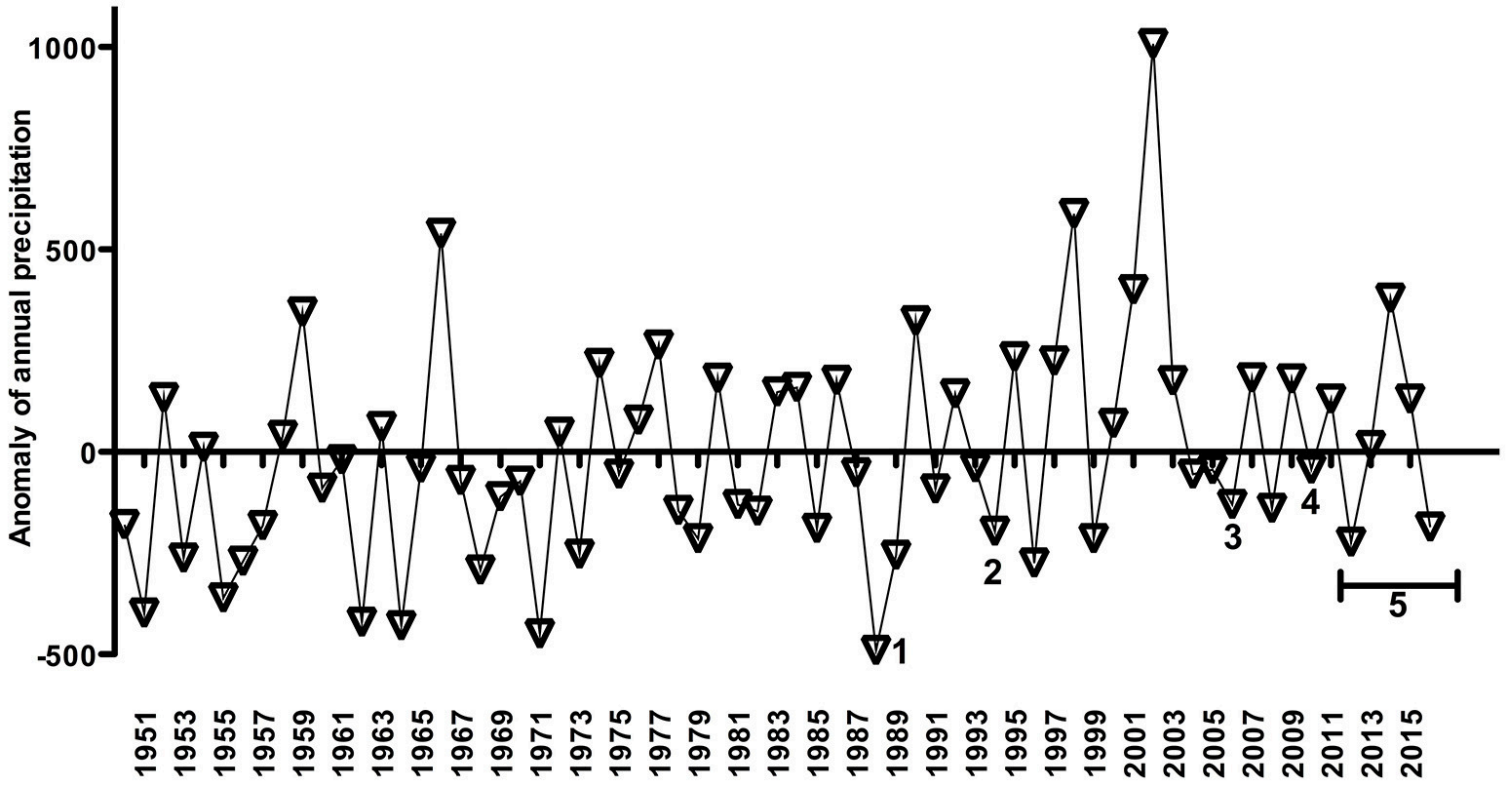
Cyanobacteria blooms or non-blooms estimates published and of this work (summers of 2012, 2013, 2014, 2015, 2016, and 2017) overlaid with an anomaly of rainfall time-series as in Figure 3B. Note that the anomaly of rainfall time-series ends in 2016. **1** refers to Odebrecht et al. (2005), and **2** Yunes et al. (1998a), **3** and **4** Rosa and Garcia (2013), and **5** refers to cyanobacteria estimates near the cities of Tapes and São Lourenço do Sul (offered by FEPAM, Brazil) (see text for more details).

Some observations or published data of MAC across the PL system were overlaid as numbers on the annual precipitation from 1950 to 2016–2017 (Figure 6). There were registered high abundances of MAC only on the negative anomaly of annual precipitation, i.e., over dry years, specifically in 1988, 1994, 2006, 2010–2012, and 2017 (Figure 6). These published data described these years associated with long water residence time and strong thermal stratification in the Patos Lagoon.

## Discussion

We have used data of air temperature, annual precipitation, and river discharge to conduct this long-term study aiming to relate cyanobacteria abundance, mainly blooms of MAC, with meteorological and hydrographic variability in the Patos Lagoon system. For the period of 2001–2017 we found somehow an association between the ENSO effect and the records of cyanobacteria concentrations >10^3^ cells mL^−1^ in particular austral summer period (January–March), mainly during La Niña and negative monthly anomalies in El Niño years. It seems that low rainfall related to negative anomalies of ENSO index could mean the MAC populations should not be quickly advected from the limnic parts of the PL system. This dynamic could make more apparent the biomass accumulation of MAC across that limnic region. However, there will be necessary further studies on hydrodynamics and water residence time in order to draw a comprehensive picture of the frequency and intensity of MAC blooms along the whole PL system. Also, we clearly identified that a monitoring program on these MAC blooms need to be implemented as these microcystin-producing species have been raised worldwide (Preece et al., 2017 and references therein). *Microcystis* blooms have been more common both in freshwater ambients and contiguous estuarine and marine systems, for instance, in the Argentinean and Uruguayan coastal regions under influence of Río de La Plata, many European nearshore aquatic systems and USA estuaries and bays (Preece et al., 2017). Coupling with climate change effects, many works stated that hydrologic alterations including intense storms and prolonged droughts followed by increased rainfalls will lead to increased freshwater inputs; and these hydrologic shifts can transport nutrients, dilute coastal receiving waters, promote cyanoHABs, and increase the occurrence of microcystins within those ambients (Paerl and Huisman, 2009; Paerl and Otten, 2013; Preece et al., 2017).

ENSO influence over the hydrological cycle on the southernmost part of Brazil is fairly well known (e.g., Odebrecht et al., 2010b). These ENSO influence and rainfall effects on phytoplankton dynamics, not specifically on cyanobacteria, have already been discussed for the years from 1993 to 2012 (e.g., Haraguchi et al., 2015). These authors observed that high rainfall and river discharge periods are associated with low phytoplankton biomass. Also, they showed that dinoflagellates and cyanobacteria presented an increasing trend in relative contribution to the phytoplankton community in the estuarine region of PL (Haraguchi et al., 2015).

Otherwise, when a longer time-series (1950–2016) is considered, we observe a significant increasing trend in rainfall, which should be interpreted distinctly in relation to the dynamics of MAC in the PL system. Although no complete time-series of MAC or other cyanobacteria is available for this whole period, we could expect that MAC blooms tended to be advected to the outlet of the PL system after and during time periods of heavy rain. However, the lack of data prevents us from affirming whether these MAC blooms had not happened in the past decades.

In summer and autumn, the growth and patch development of cyanobacteria are typically registered (Yunes, 2009), and NE wind is predominant in the region. Considering this wind pattern and the high prevalence of cyanobacteria, we suggest that cyanobacteria surface scums can be transported from the northern region to the southern estuarine portion of PL and to its adjacent coastal region. Also, some advection of cyanobacterial patches may occur to the west margin of this aquatic system where many cities are located, as seen in a LandSat8 ETM+ image for February-2017 (see Figure 1).

Since the tidal range in PL is low (amplitude 0.4 m) and the hydrodynamics is mainly controlled by the action of wind and freshwater discharge (Möller et al., 2001; Vaz et al., 2006), the NE wind pattern could partially explain the observations of high numbers of cyanobacteria near the cities of Tapes (4 × 10^4^ cells mL^−1^) and São Lourenço do Sul (2.5 × 10^4^ cells mL^−1^) in summer period of 2011–2012 (as seen at Table 1 and Figure 4). Then, coupled with high rainfall periods, NE winds should accelerate the export of MAC biomass to the coastal region. In contrast, southerly winds are more important during the winter periods, from June to September (data not shown), when the passage of frontal polar systems (Stech and Lorenzetti, 1992; Klein, 1997) cause many environmental alterations in the phytoplankton dynamics (Odebrecht et al., 2010a).

Despite our short time-series, the decreasing trend in river discharge could be associated with the years of low rainfall (see Figure 3B, 6), and are coincidental with the available high cyanobacteria estimates. As saltwater intrusion seems to increase the retention times promoting phytoplankton blooms during low runoff periods in the PL estuary (Haraguchi et al., 2015; Odebrecht et al., 2015), we might suggest that these periods could also favor the development of cyanobacteria blooms in the upper parts of the PL due to a longer retention time. As the season progresses, a part of these cyanobacterial blooms could be advected to the estuary and coastal region, mainly during high rainfall periods (Odebrecht et al., 2005), probably coupled with the NE winds. Furthermore, this kind of event has been seen during field surveys (Yunes et al., 1998a) and MAC development within the PL does depend upon salt intrusion to make phosphates available from sediments and increase the pH suitable for cyanobacteria (Yunes, 2009).

### Implications of cyanohabs (especially of MAC) and their potential effects

The earliest published data of laboratory tests of toxicity of cyanobacteria in the Patos Lagoon system dated back to late summer 1994 (Table 2), when the culture of *Microcystis aeruginosa* complex (MAC) labeled as *M. aeruginosa* RST9501 strain was initiated. The predominant species in all these blooms were always related to MAC, while other species rarely occurred. In general, those microcystin-related toxicity tests gave positive results for the crustaceans *Ceriodaphnia dubia* (Monteiro et al., 2006), *Artemia salina* (Yunes et al., 1996, 1998a) and the native estuarine species *Kalliapseudes schubartii* (Montagnolli et al., 2004) and, also toxin accumulation by the white clam *Mesodesma mactroides* (Leão et al., 2010). Among the microcystin variants detected within the Patos Lagoon system, D-Leu microcystin LR was the predominant form synthesized by those *Microcystis* blooms during summer (Matthiensen et al., 2000). As far as we know, only a biodegradation process carried by heterotrophic bacteria *Burkholderia* appeared to diminish microcystins concentration into sediments and water column (Lemes et al., 2008).

**Table 2:** Studies with *Microcystis aeruginosa* blooms and strain RST9501 isolated from the Patos Lagoon estuary from 1994 to 2012.

**Period**	**Test**	**Concentration^a^**	**References**	**Observation**
Dec-1993 to Apr-1995	Brine shrimp Brine shrimp	^a^LC_50(18h)_ 1.40 mg.mL d.w. containing 0.97 (MC) μg.mg d.w. ^a^LC_50(18h)_ 0.90 mg.mL d.w. containing (MC) 0.28 μg.mg^−1^ d.w.	Yunes et al., 1998a	Bloom samples in the Patos Lagoon estuary at 2 sites.
Oct-1995, Feb-1995 and Feb-1996	Toxin in different growth stages	^a^0.01–0.19 μg.mg^−1^ d.w.	Yunes et al., 1998b	Up to 289 μg L^−1^ MC-LR extracellularly in lagoon waters.
Dec-1994 to Aug-1995	Bloom Mice	^a^1μg.mg^−1^ d.w ^a^LD_50_ < 100 mg.kg^−1^ b.w.	Matthiensen et al., 1999	MC-LR and another analog
Apr-1997 to Jul-1998	Bloom	^a^0.1–118.2 μg.g^−1^	Minillo et al., 2000	*Microcystis* blooms are frequent annually varying on intensity.
1994–1996 Monitoring program	Salinity and growth	MC concentration is inversely proportion of salinity increase up to 4.	Salomon et al., 2001	Strain isolated from Patos Lagoon estuary (Laboratório de Cianobactérias e Ficotoxinas, LCF).
Laboratory test	Salinity and growth	Cell growth affected by low salt concentration.	Lima and Yunes, 2003	2 g of marine salt is enough to inhibit the growth of a 2 L of *M. aeruginosa* culture.
Laboratory test	Lagoon sediment, *Kalliapseudes schubartii*	^a^LC_50(96h)_ 1.44 mg. mL^−1^	Montagnolli et al., 2004	Sediments spiked with the toxic lyophilized material of the strain RST9501 to *K. schubartii*.
Oct-1997 to Apr-1998	*Microcystis* blooms	69,000–100,000 colonies L^−1^	Ferreira et al., 2004	Bloom samples collected in the Patos Lagoon
Laboratory test	*Ceriodaphnia dubia*	Inhibition of *C. dubia*, reproduction, and growth. Although, NPJT–O1 strain has no toxicity to mice.	Monteiro et al., 2006	The presence of the two strains of *M. aeruginosa* inhibited both reproduction and growth in *C. dubia*, regardless the presence of microcystin.
Feb-2006	Bloom	0.14 μg (MC) L^−1^	Laboratório de Cianobactérias e Ficotoxinas (J.S. Yunes, personal communication)	São Lourenço do Sul bloom
Laboratory test	Estuarine and coastal water samples from Patos Lagoon	Biodegradation rate of exponential decay inside and outside the estuary were similar. A microcystin degradative bacterium was isolated from the estuarine region. Partial sequence of the 16S rDNA showed 96% homology with the *Burkholderia* genus.	Lemes et al., 2008	Spiked with purified and semi-purified MC-LR and [D-Leu^−1^] MC-LR has show growth of heterothophic aquatic bacteria, from Patos lagoon waters.
1987–2008	Bloom	MC-LR and [D-Leu^−1^] MC-LR	Yunes, 2009	Review of last 20 years of Patos Lagoon blooms.
Laboratory test	*Mesodesma mactroides*	5.27 ± 0.23 μg.g^−1^ (dry hepatopancreas weight).	Leão et al., 2010	Clams were exposed to live cells and increasingly uptake the toxic cyanobacterium for 12 days.
2005–2012	Summer blooms	Massive bloom	Rosa and Garcia, 2013	*M. aeruginosa*, massive bloom in 2010.
Laboratory test	Carp *Cyprinus carpio*	25 μg.Kg^−1^ b.w. 50 μg.Kg^−1^ b.w.	Amado et al., 2011	Effects of aqueous extract of the cyanobacterium producing microcystin RST9501 on detoxification capacity and glutathione (GSH) synthesis in liver, brain, gill, and muscle.
Phylogenetic analysis	[D-Leu^1^] MC-LR	*Nostoc, Phormidium* produced a new microcystin that was identified as [Met^−1^] MC-LR.	Shishido et al., 2013	A convergence study toward the origins of the DNA transcript, which codes for [D-Leu^1^] MC-LR synthesis.

a *Toxicity tests performed with lyophilized bloom samples. b.w., body weight; d.w., dry weight; MC, microcystin*.

*Microcystis* might outcompete other phytoplankton species under nutrient-depleted waters (e.g., poor in Si) and within the stronger thermal-stratified water column, which are conditions normally found in the summer period (Paerl, 2017 and references therein). However, this scenario of the nutrient-limited condition should not be the case of the PL system (Odebrecht et al., 2015), where light has been considered the main limiting factor for phytoplankton growth (Abreu et al., 2016). Particularly, MAC has proved to display a fairly complex life cycle with different colony sizes within the PL (Yunes et al., 1996) and in accordance to the literature (Reynolds et al., 1981), which can add to their fitness within a varying light ambient.

Moreover, the majority of works related to MAC in the PL focused on its potential toxicity with environmental and human health impacts (Yunes, 2009 and references therein). Likewise, those works compiled in Table 2 pointed out that the massive blooms formed in pre-limnic and limnic waters of the PL would exert their potential noxious effects depending on the wind-driven hydrodynamics as described here. These blooms can even be an undesirable feature in human activities, such as fishing and leisure, along with the beaches and cities of PL margins (Yunes, 2009).

In short, we can suggest that these MAC blooms will tend to be more critical during low rainfall periods and/or negative summer anomalies seen in the 3.4-ENSO index (December to February, at least, but appearing up to April-May next year). At the same time, the westward NE wind-associating advection of these potentially toxic, cyanobacterial patches can be a serious threat to human health at the margins of some cities. Otherwise, heavy rainfall precipitation/positive anomalies of the 3.4-ENSO index should alleviate the permanence of these MAC blooms near these cities. As well, there should be an export of organic suspended material and cyanotoxins to the estuary of Patos Lagoon and to the immediate coastal region.

## Author contributions

MS analyzed all dataset and wrote the manuscript. JM supervised all the work and co-discussed the interannual variability and climate change issues. LC was responsible for the review of toxicological studies and revised the text. EK co-analyzed the meteorological dataset and revised the text. JY supervised all the work and revised the text.

### Conflict of interest statement

The authors declare that the research was conducted in the absence of any commercial or financial relationships that could be construed as a potential conflict of interest.

## References

[B1] Abreu P. C. Marangoni J. Odebrecht C. (2016). So close, so far: differences in long-term chlorophyll *a* variability in three nearby estuarine-coastal stations. Mar. Biol. Res. 13, 9–21. 10.1080/17451000.2016.1189081

[B2] Amado L. L. Garcia M. L. Ramos P. B. Yunes J. S. Monserrat J. M. (2011). Influence of a toxic *Microcystis aeruginosa* strain on glutathione synthesis and glutathione-S-transferase activity in common carp *Cyprinus carpio* (Teleostei: Cyprinidae). Arch. Environ. Contam. Toxicol. 60, 319–326. 10.1007/s00244-010-9594-2 20809345

[B3] Battye W. Aneja V. P. Schlesinger W. H. (2017). Is nitrogen the next carbon? Earth Fut. 5, 894–904. 10.1002/2017EF000592

[B4] Cloern J. E. Abreu P. C. Carstensen J. Chauvaud L. Elmgren R. Grall J.. (2016). Human activities and climate variability drive fast-paced change across the world's estuarine-coastal ecosystems. Glob. Chang. Biol. 22, 513–529. 10.1111/gcb.13059 26242490

[B5] Ferreira A. H. F. Minillo A. Silva L. M. Yunes J. S. (2004). Ocorrência de *Anabaena spiroides* (Cianobactéria) no estuário da Lagoa dos Patos (RS, Brasil) no verão-outono de 1998. Atlântica 26, 17–26.

[B6] Haraguchi L. Carstensen J. Abreu P. C. Odebrecht C. (2015). Long-term changes of the phytoplankton community and biomass in the subtropical shallow Patos Lagoon Estuary, Brazil. Estu. Coast. Shelf Sci. 162, 76–87. 10.1016/j.ecss.2015.03.007

[B7] IPCC (2014). Climate Change 2014: Synthesis Report, in Contribution of Working Groups I, II and III to the Fifth Assessment Report of the Intergovernmental Panel on Climate Change, eds Pachauri R. K. Meyer L. A. (Geneva: IPCC), 151.

[B8] Klein A. H. F. (1997). Regional climate, in Subtropical Convergence Environments, eds Seeliger U. Odebrecht C. Castelo J. P. (Berlin: Springer-Verlag), 5–7.

[B9] Kleinteich J. Puddick J. Wood S. A. Hildebrand F. Laughinghouse I. V. Pearce D. A.. (2018). Toxic cyanobacteria in Svalbard: chemical diversity of microcystins detected using a liquid chromatography mass spectrometry precursor ion screening method. Toxins 10:147. 10.3390/toxins10040147 29614044 PMC5923313

[B10] Komárek J. Kastovsky J. Mares J. Johansen J. R. (2014). Taxonomic classification of cyanoprokaryotes (cyanobacterial genera) 2014, using a polyphasic approach. Preslia 86, 295–335.

[B11] Leão J. C. Giordano S. B. Yunes J. S. (2010). Microcystins uptake by the yellow clam *Mesodesma mactroides* (Bivalvia, Mactroidea). Atlântica 32, 79–85. 10.5088/atl.2010.32.1.79

[B12] Lehman P. W. Boyer G. Hall C. Waller S. Gehrts K. (2005). Distribution and toxicity of a new colonial *Microcystis aeruginosa* bloom in the San Francisco Bay Estuary, California. Hydrobiologia 541, 87–99. 10.1007/s10750-004-4670-0

[B13] Lemes G. A. Kersanach R. Pinto L. S. P. Dellagostin O. A. Yunes J. S. Matthiensen A. (2008). Biodegradation of microcystins by aquatic *Burkholderia* sp. from a south Brazilian coastal lagoon. Ecotoxicol. Environ. Saf. 69, 358–365. 10.1016/j.ecoenv.2007.03.013 17531317

[B14] Lima J. B. Yunes J. S. (2003). Efeito do sal marinho granulado no controle de cianobactérias nocivas em mananciais. Vetor 13, 27–35.

[B15] Matthiensen A. Beattie K. A. Yunes J. S. Kaya K. Codd G. A. (2000). [D-Leu^1^] Microcystin-LR, from the cyanobacterium *Microcystis* RST 9501 and from a *Microcystis* bloom in the patos lagoon estuary, Brazil. Phytochemistry 55, 383–387. 10.1016/S0031-9422(00)00335-6 11140597

[B16] Matthiensen A. Yunes J. S. Codd G. A. (1999). Ocorrência, distribuição e toxicidade de cianobactérias no estuário da Lagoa dos Patos, RS. Rev. Bras. Biol. 59, 361–376. 10.1590/S0034-71081999000300002 10765462

[B17] Minillo A. Ferreira A. H. F. Yunes J. S. (2000). Detecção de microcistina em florações de *Microcystis aeruginosa* no estuário da Lagoa dos Patos entre 1997 e 1998. Atlântica 22, 81–93.

[B18] Möller O. O. Castaing P. Salomon J. C. Lazure P. (2001). The influence of local and non-local forcing effects on the subtidal circulation of Patos Lagoon. Estuaries 24, 297–311. 10.2307/1352953

[B19] Montagnolli W. Zamboni A. Luvizotto-Santos R. Yunes J. S. (2004). Acute effects of *Microcystis aeruginosa* from the Patos Lagoon estuary, Southern Brazil, on the microcrustacean *Kalliapseudes schubartii* (Crustacea: Tanaidacea). Arch. Environ. Contam. Toxicol. 46, 463–469. 10.1007/s00244-003-2304-6 15253043

[B20] Monteiro N. J. C. Yunes J. S. Bohrer-Morel M. B. (2006). Effects of the *Microcystis aeruginosa* strain RST9501 from Patos Lagoon, RS, on growth and reproduction of the cladoceran *Ceriodaphnia dubia*. J Brazil. Soc. Ecotoxicol. 1, 93–96. 10.5132/jbse.2006.01.020

[B21] Niencheski L. F. Baumgarten M. G. Z. (2007). Water quality in Mangueira Bay: anthropic and natural contamination. J. Coast. Res. 47, 56–62. 10.2112/1551-5036-47.sp1.56

[B22] Odebrecht C. Abreu P. C. Bemvenuti C. E. Coppertino M. Muelbert J. H. Vieira J. P. (2010a). The Patos Lagoon Estuary: biotic responses to natural and anthropogenic impacts in the last decades (1979-2008), in Coastal Lagoons: Systems of Natural and Anthropogenic Change, eds Kennisch M. Paerl H. (Boca Raton, Fl: Taylor & Francis/CRC Press), 437–459.

[B23] Odebrecht C. Abreu P. C. Carstensen J. (2015). Retention time generates short-term phytoplankton blooms in a shallow microtidal subtropical estuary. Estuar. Coast. Shelf Sci. 162, 35–44. 10.1016/j.ecss.2015.03.004

[B24] Odebrecht C. Abreu P. C. Möller O. O. Jr. Niencheski L. F. H. Proença L. A. Torgan L.C. (2005). Drought effects on pelagic properties in the shallow and turbid Patos Lagoon, Brazil. Estuaries 28, 675–685. 10.1007/BF02732906

[B25] Odebrecht C. Bergesch M. Rörig L. R. Abreu P. C. (2010b). Phytoplankton interannual variability at Cassino Beach, southern Brazil (1992–2007), with emphasis on the surf-zone diatom *Asterionellopsis glacialis*. Estu. Coasts. 33, 570–583. 10.1007/s12237-009-9176-6

[B26] Odebrecht C. Selliger U. Coutinho R. Torgan L. C. (1987). Florações de Microcystis (cianobactérias) na Lagoa dos Patos, RS, in Proceedings of the 1st Simpósio sobre Ecossistemas Costeiros Sul e Sudeste do Brasil: Síntese do Conhecimento, ed ACIESP (Águas de Lindóia: ACIESP), 11–16.

[B27] Paerl H. W. (2017). Controlling harmful cyanobacterial blooms in a climatically more extreme world: management options and research needs. J. Plankton Res., 39, 763–771. 10.1093/plankt/fbx042

[B28] Paerl H. W. Huisman J. (2009). Climate change: a catalyst for global expansion of harmful cyanobacterial blooms. Environ. Microbiol. Rep. 1, 27–37. 10.1111/j.1758-2229.2008.00004.x 23765717

[B29] Paerl H. W. Otten T. G. (2013). Harmful cyanobacterial blooms: causes, consequences, and controls. Microb. Ecol. 65, 995–1010. 10.1007/s00248-012-0159-y 23314096

[B30] Paerl H. W. Pinckney J. L. (1996). A mini-review of microbial consortia: their roles in aquatic production and biogeochemical cycling. Microb. Ecol. 31, 225–247. 10.1007/BF00171569 8661534

[B31] Preece E. P. Hardy F. J. Moore B. C. Bryan M. (2017). A review of microcystin detections in estuarine and marine waters: environmental implications and human health risk. Harmful Algae 61, 31–45. 10.1016/j.hal.2016.11.006

[B32] Reynolds C. S. Jaworski G. H. M. Cmiech H. A. Leedale G. F. (1981). On the annual cycle of the blue-green alga *Microcystis aeruginosa* Kutz. emend. Elenkin. Philos. Trans. R. Soc. B. 293, 419–477. 10.1098/rstb.1981.0081

[B33] Rosa V. C. Garcia M. (2013). Ocorrência de *Ulva* spp., *Polysiphonia* sp., e *Microcystis aeruginosa* nas praias do Saco do Laranjal, Pelotas, RS. Rev. Thema 10, 122–137. 10.15536/thema.10.2013.122-137.127

[B34] Salomon P. S. Yunes J. S. Matthiensen A. Codd G. A. (2001). Chapter 16: Does salinity affect the toxin content of an estuarine strain of *Microcystis aeruginosa*?,in Mycotoxin and Phycotoxin in Perspective at the Turn of the Millennium eds Koe W. J. Samson R. Van Egmond H. P. Gilberto J. Sabino M. (Holand: IUPAC/AOAC), 537–548.

[B35] Shishido T. K. Kaasalainen U. Fewer D. P. Rouhiainen L. Jokela J. Wahlsten M.. (2013). Convergent evolution of [D-Leucine1] microcystin-LR in taxonomically disparate cyanobacteria. BMC Evol. Biol. 13:86. 10.1186/1471-2148-13-86 23601305 PMC3640908

[B36] Stech J. L. Lorenzetti J. A. (1992). The response of the south Brazil bight to the passage of wintertime cold fronts. J. Geophys. Res., 97, 9507–9520. 10.1029/92JC00486

[B37] Suikkanen S. Pulina S. Engström J. Lehtiniemi M. Lehtinen S. Brutemark A. (2013). Climate change and eutrophication induced shifts in northern summer plankton communities. PLoS ONE 8:e66475. 10.1371/journal.pone.0066475 23776676 PMC3680480

[B38] Vaz A. C. Möller O. O. Jr de Almeida T.L. (2006). Análise quantitativa da descarga dos rios afluentes da Lagoa dos Patos. Atlântica 28, 13–23.

[B39] Yunes J. S. (2009). Florações de *Microcystis* na Lagoa dos Patos e o seu estuário: 20 anos de estudos. Oecol. Brasil. 13, 313–318. 10.4257/oeco.2009.1302.06

[B40] Yunes J. S. Matthiensen A. Parise M. Salomon P. S. Raggett S. L. Beattie K. A. (1998a). Microcystis aeruginosa growth stages and the occurrence of microcystins in PatosLaggon, Southern Brazil, in Xunta de Galícia and Intergovernmental Oceanographic Commission of UNESCO, eds Reguera B. Blanco J. Fernández M. L. Wyatt T. (Vigo: Harmful Algae), 18–21.

[B41] Yunes J. S. Niencheski L. F. H. Salomon P. S. Parise M. Beattie K. A. Raggett S. (1998b). Effect of nutrient balance and physical factors on blooms of toxic cyanobacteria in Patos Lagoon, southern Brazil. Verhand. Int. Verein Limnol. 26, 1796–1800. 10.1080/03680770.1995.11901048

[B42] Yunes J. S. Salomon P. S. Matthiensen A. Beattie K. A. Raggett S. L. Codd G. A. (1996). Toxic blooms of cyanobacteria in the Patos Lagoon Estuary, southern Brazil. J. Aqua. Ecosys. Health 5, 223–229. 10.1007/BF00662183

